# Supplementary figures and images for: Thymoquinone-Loaded Chitosan Nanoparticles as Natural Preservative Agent in Cosmetic Products
Source: Int J Mol Sci. 2022 Jan 14;23(2):898. doi: 10.3390/ijms23020898 (PMC8778794; doi:10.3390/ijms23020898)

**Figure S1.** FTIR of TQ, NPCH and NPCH-TQ

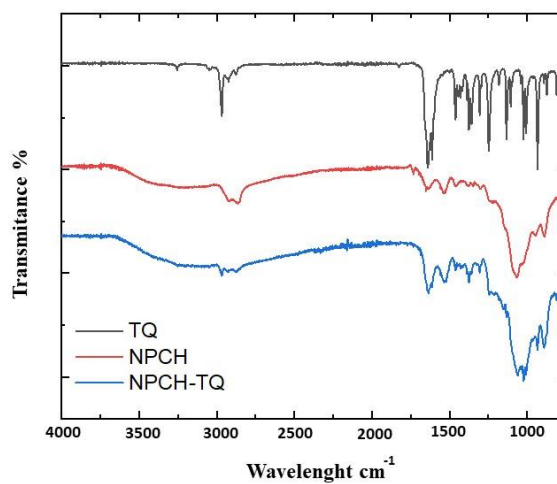

**Figure S2.** DSC and TGA of TQ (a) and NPCH-TQ (b)

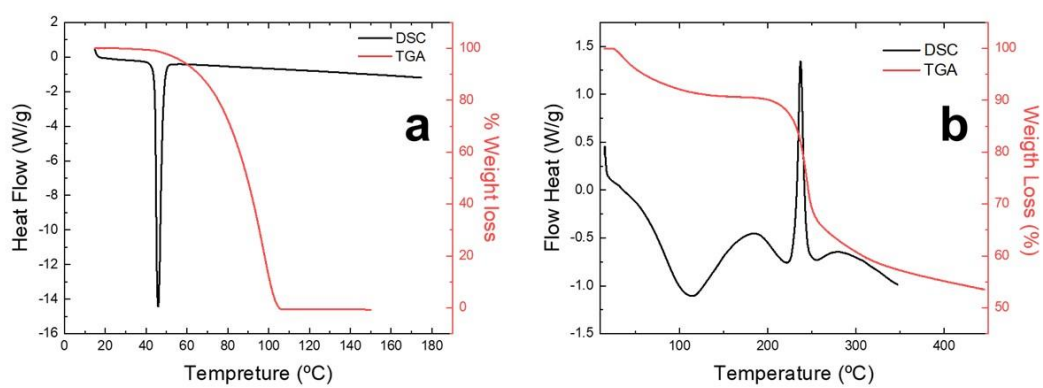

Supplement: Supplementary file 1 [file ijms-23-00898-s001.zip › ijms-1547035-supplementary.pdf]
